# Protoplasmic astrocytoma with multifocal involvement: case report and radiological findings

**DOI:** 10.1259/bjrcr.20150057

**Published:** 2015-05-26

**Authors:** A Abdullah, P Entezami, L Halpin, J Feldmeier, R E Mrak, D Gaudin

**Affiliations:** ^1^Department of Radiology, University of Toledo Medical Center, Toledo, OH, USA; ^2^Division of Neurosurgery, Department of Surgery, University of Toledo Medical Center, Toledo, OH, USA; ^3^Department of Radiation Oncology, University of Toledo Medical Center, Toledo, OH, USA; ^4^Department of Pathology, University of Toledo Medical Center, Toledo, OH, USA

## Abstract

Protoplasmic astrocytomas are a poorly characterized and extremely rare subtype of astrocytoma. We describe the CT, MR and ^18^F-fludeoxyglucose positron emission tomography (FDG-PET) findings of a multifocal protoplasmic astrocytoma in a 29-year-old male with neurological deficits. He was initially diagnosed with neurosarcoidosis based on imaging. MRI demonstrated intraparenchymal lesions involving the right temporal lobe and cerebellum. These appeared as extremely hyperintense signals on *T*_2_ weighted imaging and as homogeneous enhancements with a small non-enhancing cystic component on contrast-enhanced MR. Diffuse post-contrast enhancement of the craniospinal meninges was also noted. Post-radiation therapy PET-CT demonstrated a highly FDG-avid tumour in the right temporal lobe and left cerebellum. To our knowledge, a multifocal form of protoplasmic astrocytoma in an adult patient has not been previously described.

Astrocytomas are the most prevalent type of primary central nervous system (CNS) neoplasm. They are classified by the World Health Organization (WHO) according to histological grade. Low-grade astrocytomas (WHO grade II) are diffusely infiltrating tumours, and can be further subdivided into fibrillary, gemistocytic and protoplasmic subtypes.^1,2^ Prognosis is known to correlate to histology, with fibrillary and protoplasmic subtypes carrying a better prognosis than do gemistocytic variants.^3^

In aggregate, all three forms of diffuse astrocytomas account for roughly 5% of primary CNS tumours and 10–15% of astrocytic gliomas, with protoplasmic being one of the rarest forms.^3–5^ There are many diagnostic challenges in the recognition of this entity, owing to both the non-specific preoperative radiological findings as well as a paucity of literature describing it.^2, 6–8^ Protoplasmic astrocytomas are composed of cells that resemble protoplasmic astrocytes and share several features with the other low-grade astrocytomas, including low cellularity and mitotic activity, and sparse nuclear atypia. Additionally, neovascularization and necrosis are absent.^4,5^ Compared with other grade II astrocytomas, protoplasmic types have a tendency to present at a younger age and have a greater propensity to involve the temporal lobe.

Although glioblastomas (WHO grade IV) have been known to have multiple foci, an adult patient with multifocal grade II disease involving multiple lesions in both the brain and the spine has not yet been described. We report such a case, with an emphasis on the CT, MR and ^18^F-fludeoxyglucose positron emission tomography (FDG-PET) imaging characteristics of this extremely rare presentation. The multifocal nature of this patient’s low-grade astrocytoma—including involvement of the temporal lobe, posterior fossa and craniospinal meninges—makes this case both unique and significant for the interpretation of the radiological findings associated with adult CNS tumours.

## Case report

A 29-year-old male presented with progressively worsening symptoms, including altered mental status, lower extremity weakness, nausea and vomiting for 6 months, with symptomatology extending back more than 6 years prior to this decline. He also reported headaches, dizziness, multiple falls, and right facial numbness and tingling during his 6-month decline. Previously, he had had seizures that were controlled with medication. Prior to referral to neurosurgery, his presumptive diagnosis was hydrocephalus, for which he had a ventriculoperitoneal shunt placed. Neurological examination was significant for mild dysarthria, a non-reactive left pupil, nystagmus on primary gaze of the left eye, bilateral nystagmus on horizontal gaze and mild action tremors in both hands.

**Figure 1.**
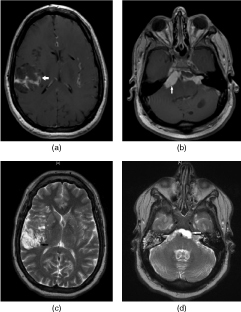
Contrast-enhanced *T*_1_ weighted MRI with a solid cystic mass in the (a) right temporal lobe and (b) right side of the prepontine cistern with an associated cystic component extending towards the midline; (c,d) *T*_2_ weighted imaging of lesions demonstrated extremely high signal intensity. Arrows emphasize the locations of lesions.

Initial pre- and post-contrast-enhanced CT scans demonstrated diffuse enhancement of the basimeninges as well as heterogeneously enhancing cystic and solid lesions in the right temporal lobe and left cerebellum. Subsequent contrast-enhanced *T*_1_ weighted MRI of the brain demonstrated extensive post-contrast nodular enhancement of the basimeninges associated with an approximately 5 × 4 cm solid and cystic mass in the right temporal lobe (Figure 1a). An additional elongated enhancing mass was seen on the right side of the prepontine cistern measuring 2.3 × 1.1 cm in size, with an associated cystic component that extended towards the midline from this mass (Figure 1b). The left cerebellum was also involved, with a solid, enhancing mass that crossed the midline. On *T*_2_ weighted imaging, the cystic components of these lesions demonstrated extremely high signal intensity (Figure 1c,d) that was suppressed on fluid-attenuated inversion-recovery (FLAIR) imaging, while *T*_1_ weighted imaging demonstrated hypointense signal.

Initial laboratory findings were significant only for an elevated erythrocyte sedimentation rate and C-reactive protein. CT of the chest was performed, demonstrating no evidence of pulmonary sarcoidosis, and subsequent biopsies of the right temporal lobe and dura were negative for neoplasm or any other abnormality. In addition, analyses of aspirated cyst contents were negative for inflammation or malignancy. A presumptive diagnosis of neurosarcoidosis was considered and the patient was started on prednisone. He had symptomatic improvement with the prednisone and was discharged following recovery from the biopsy.

**Figure 2.**
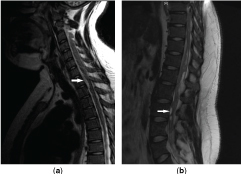
MRI views of the spinal axis showing diffuse nodular meningeal enhancement of the (a) cervical and (b) lumbar spine. Arrows emphasize the locations of lesions.

**Figure 3.**
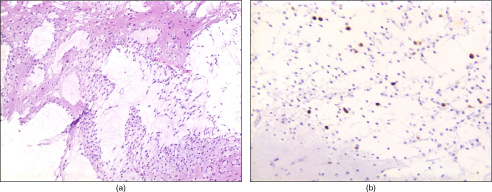
Histopathology showing (a) astrocytoma with protoplasmic features and (b) Ki-67 immunohistochemistry with labelling rate of approximately 5% suggestive of more aggressive behaviour.

Follow-up MRI 11 months later demonstrated progression of his disease with an interval increase in size of the intracranial lesions. Views of the spinal axis demonstrated diffuse nodular meningeal enhancement (Figure 2). The patient underwent a repeat ultrasound-guided brain biopsy. This time, histopathological analysis of the biopsy specimen showed an astrocytoma with protoplasmic features (Figure 3a). Immunohistochemistry for Ki-67 (a cell proliferation marker) showed a labelling rate of approximately 5%, which was somewhat higher than the 0–2% commonly seen in low-grade tumours, suggestive of more aggressive behaviour (Figure 3b).^5^

Owing to the multicentric nature of the tumour, craniospinal radiation therapy was recommended instead of radical surgical resection. After multiple cycles of radiation therapy with cumulative doses totalling 1800 cGy to the brain and 1620 cGy to the spine, the patient underwent repeat MRI. The studies showed a slight interval increase in size of the brain lesions but decrease in nodular enhancement of the spinal meninges. A whole body PET-CT was performed to assess disease status and demonstrated a standard uptake value of 13 in the right temporal lesion and 12 in the left cerebellar lesion, which was consistent with a highly metabolically active tumour. No definite increase in uptake was seen in the spinal region or elsewhere.

**Figure 4.**
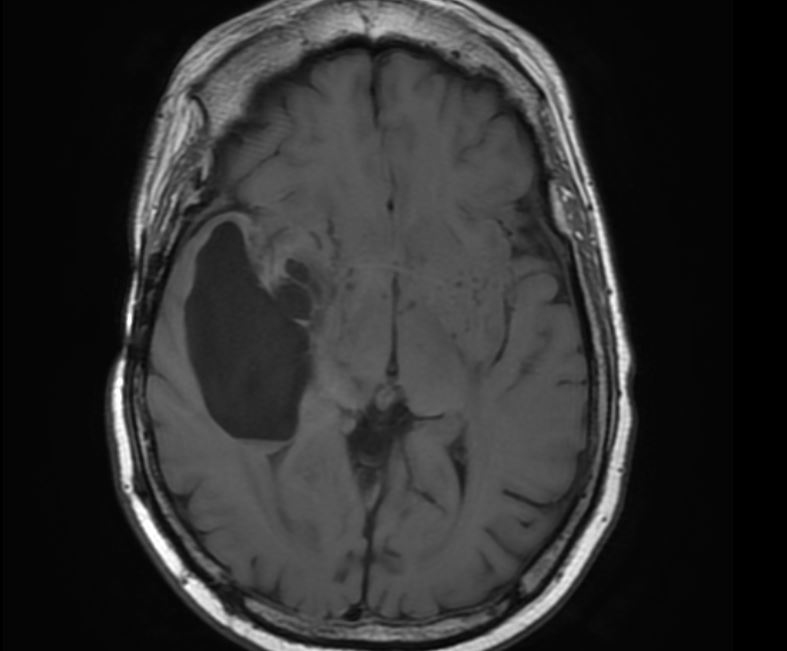
Axial *T*_1_ weighted MRI showing disease progression, with enlargement of the cystic component of the temporal lobe lesion.

Following treatment, the patient continued to take dexamethasone for symptomatic relief. He reported a much improved condition despite mild difficulty ambulating, short-term memory loss, occasional euphoria and swollen tongue. However, his disease progressed (Figure 4) and did not respond to further medical or surgical management, and he ultimately passed away.

## Discussion

There are two considerations regarding the pathological diagnosis in the current study. Ordinary diffuse astrocytomas (WHO grade II) may have foci with protoplasmic features; thus, it is important to demonstrate that the entire tumour has these features before making a diagnosis of protoplasmic astrocytoma. In the present case, we had only one small (and superficial) biopsy confirming the diagnosis, and for this reason, the diagnosis of "astrocytoma with protoplasmic features" was made instead of "protoplasmic astrocytoma". Although no biopsy was obtained from the spinal lesions, it was determined that these lesions were of similar aetiology to the biopsy specimen taken from the brain.

A 1995 study of 16 purely protoplasmic astrocytomas more frequently observed the tumours in males at a mean age of 20.7 years.^9^ The duration of symptoms ranged from 7 months to 28 years, and all patients presented with seizures. None of the tumours in this study was multifocal in nature and many underwent complete resection. Of the nine patients who were managed surgically, only one had a recurrence.^9^

In a recent series, Tay et al^10^ reviewed the MR images and histopathology of eight consecutive cases of biopsy-proven protoplasmic astrocytomas. They concluded that a primary cerebral neoplasm representing a protoplasmic astrocytoma should be considered as a differential for large frontal or temporal tumours with increased signal on *T*_2_ weighted imaging and a large proportion of the tumour demonstrating substantial *T*_2_ FLAIR suppression. In our patient, the intraparenchymal brain lesions demonstrated similar findings on *T*_2_ weighted and FLAIR MR. However, we observed avid enhancement of the solid components of these lesions in contrast to the findings reported by Tay et al^10^ that demonstrated none-to-moderate post-contrast enhancement. Furthermore, the intraparenchymal cranial lesions in our case had a large solid component, whereas prior studies included, at most, a minimal nodular component. The leptomeningeal involvement in our patient showed significant post-contrast enhancement and nodular areas of high *T*_2_ weighted signal intensity involving the craniospinal meninges and the cauda equina, which is also novel.

FDG-PET imaging of protoplasmic tumours has not been previously reported in literature. In our patient, the intraparenchymal cranial lesions demonstrated avid FDG uptake, which may correlate with the somewhat aggressive nature of the neoplasm observed histologically.

## Conclusions

Low-grade astrocytomas are slow-growing and well-differentiated tumours, although there is a tendency for malignant progression that is often refractory to surgical treatment, chemotherapy or radiation therapy. Protoplasmic astrocytomas are a rare variant of diffuse astrocytomas with a generally favourable prognosis. Radiologically, these tumours can mimic neurosarcoidosis, Wegener’s, tuberculosis, leukaemia, lymphoma, primary CNS neuromatosis, metastases and, less commonly, infections, among other rare entities.^6^ This is further complicated by the fact that sarcoidosis presents with neurological involvement at initial presentation in 5–10% of the individuals.^4^

Our patient’s presentation and clinical course demonstrates the importance of obtaining biopsies in cases of diffuse meningeal enhancement on MRI when a diagnosis is unknown and clinical history supports multiple differential diagnoses. Radiologists and neurosurgeons need to be aware of protoplasmic astrocytomas, as the non-specific radiological and clinical presentations can make this a complex and challenging diagnosis.

## Learning points

1. Protoplasmic astrocytomas are a poorly characterized and extremely rare subtype of astrocytoma. Multifocal variants of this tumour have seldom been reported.
2. Radiologically, these tumours can mimic neurosarcoidosis, Wegener’s, tuberculosis, leukaemia, lymphoma, primary CNS neuromatosis, metastases and, less commonly, infections, among other rare entities.
3. The non-specific radiological presentation makes obtaining a diagnosis difficult, highlighting the need for obtaining a biopsy in cases of diffuse meningeal enhancement on MRI when a diagnosis is unknown.
